# Synthesis and Characterisation of Core–Shell Microparticles Formed by Ni-Mn-Co Oxides

**DOI:** 10.3390/molecules29122927

**Published:** 2024-06-20

**Authors:** Javier García-Alonso, Svitlana Krüger, Bilge Saruhan, David Maestre, Bianchi Méndez

**Affiliations:** 1 Departamento de Física de Materiales, Facultad de CC. Físicas, Universidad Complutense de Madrid, 28040 Madrid, Spain; jgarci13@ucm.es (J.G.-A.);; 2 Institute of Materials Research, German Aerospace Center (DLR e.V.), Linder Hoehe, 51147 Cologne, Germany

**Keywords:** microparticles, core–shell, oxides

## Abstract

In this work, core and core–shell microparticles formed by Ni-Mn-Co oxides with controlled composition were fabricated by an oxalate-assisted co-precipitation route, and their properties were analysed by diverse microscopy and spectroscopy techniques. The microparticles exhibit dimensions within the 2–6 μm range and mainly consist of NiO and NiMn_2_O_4_, the latter being promoted as the temperature of the treatment increases, especially in the shell region of the microparticles. Aspects such as the shell dimensions, the vibrational modes of the spinel compounds primarily observed in the shell region, the oxidation states of the cations at the surface of the microparticles, and the achievement of a Ni-rich 811 core and a Mn-rich 631 shell were thoroughly evaluated and discussed in this work.

## 1. Introduction

Among the versatile transition metal oxides (TMOs), ternary compounds with a spinel structure (AB_2_O_4_) are gaining increasing attention in diverse fields of research such as catalysis, sensing, and, recently, energy storage systems [1,2,3]. In this AB_2_O_4_ spinel structure, A and B cations are distributed over tetrahedral and octahedral sites in a closely packed oxygen ion lattice, leading to normal or inverse spinel compounds with mixed cationic valence states [4]. Either alone or in combination with other compounds, these spinel materials demonstrated relevant physicochemical properties based on their characteristically mixed valent cations, which make them go-to materials in diverse technologies. In particular, some of these spinel oxides based on Ni, Mn, and Co are considered cornerstone materials for electrodes in Li-ion batteries (LiBs), as their combination with Li can lead to the formation of Li(Ni_x_Mn_y_Co_z_)O_2_ compounds, known as NMCs, with paramount relevance in the development of highly efficient LiBs [5,6]. In the pursuit of optimised response and wider applicability beyond the field of energy storage, challenges should be overcome for the achievement of Ni-Mn-Co oxide micro- and nanostructures with tailored morphology, dimensions, composition, and properties by means of appropriate synthesis routes and novel material design concepts. As an example, core–shell particles based on Ni-Mn-Co oxides with a Ni-rich core, Mn-rich shell, and reduced Co content play a crucial role as innovative cathode materials in LiBs with improved electrochemical performance [7,8,9]. In particular, cathodes with an 811 Ni-Mn-Co core composition are one of the most commonly employed types in high-performance Li-ion batteries due to their high energy density and enhanced storage capacity provided by the increased presence of nickel. On the other hand, the formation of the Ni^2+^ oxidation state during the repeated charge/discharge, which causes chemical and structural deterioration and, as a result, poor cyclic performance, can be addressed through the introduction of manganese (for instance, as a shell with a high manganese composition, e.g., 631), which acts not only as a stabiliser but also prevents nickel oxidation and thus reduces the risk of capacity fading. In addition, combined Ni-Mn-O spinel oxides with NiO or other binary oxides in the form of heterostructures are also gaining increasing relevance in the fields of sensing, photocatalysis, and supercapacitors [10,11,12,13].

However, the study of combined Ni-Mn-Co oxides is not straightforward, as in most cases, AB_2_O_4_ spinel oxides based on Ni, Mn, and Co, such as NiMn_2_O_4_ and MnCo_2_O_4_, exhibit similar properties, which hinders their identification and analysis by diverse techniques, thus requiring a detailed analysis in order to avoid possible misidentifications.

The applied synthesis route for such oxides has a great influence in the single-phase formation of multiple element oxides and therefore must be selected carefully to achieve the desired phase sequence. Diverse synthesis routes, including solid phase [14], co-precipitation [15,16], sol–gel [17,18], and spray-drying [19], have been reported so far for the fabrication of Ni-Mn-Co oxides with controlled composition. The issue of the non-uniformity of the reaction during solid-phase synthesis can be addressed in wet chemical methods due to the precise control of the stochiometric ratio and the mixing of raw materials at the atomic level. For instance, controlling synthesis parameters (e.g., raw material concentration, pH values, stirring speed, reaction temperature) in the case of the co-precipitation method can allow for the formation of the desired morphologies with a uniform particle size distribution using water-soluble raw materials, which are easy to handle [20]. Moreover, the co-precipitation approach provides the advantage of material production in the most simple, fast, and inexpensive conditions, making it an efficient synthesis method. Regarding the synthesis of core–shell-structured powders, a number of synthesis approaches have been applied so far, including the hydrothermal method [21], the self-assembly process [22], and the template method [23]. Among the developed methods, the co-precipitation route is also more suitable for the synthesis of core–shell-structured microparticles due to the unique growth mechanism of spherical precursors [24,25,26]. In this case, the precipitating agent has a significant effect on the properties of crystal particles and on the formation of core–shell morphologies. For instance, hydroxide-based, carbonate-based, and oxalate-assisted co-precipitation techniques are used, among which oxalic acid as a precipitator leads to the formation of fine-sized particles with good homogeneity due to the reducing properties of oxalate ions, and it is more environmentally friendly than inorganic bases, as well as being cheaper. Considering lithium battery applications, the co-precipitation route also gives rise to the possibility of synthesising spherical core–shell-structured particles with high energy densities [24] and different core and shell compositions by applying a two-stage process with the encapsulation of the core by the shell in the second step.

In this work, microparticles based on Ni-Mn-Co oxides with controlled dimensions and compositions and a core–shell structure were synthesised via a cost-efficient and easily controlled oxalate-assisted co-precipitation route and characterised by X-ray diffraction (XRD), scanning electron microscopy (SEM), energy-dispersive X-ray spectroscopy (EDS), Raman spectroscopy, and X-ray photoelectron spectroscopy (XPS) at a synchrotron facility. A list of samples and details on the synthesis route and the characterization techniques are provided in the “Materials and Methods” section.

## 2. Results and Discussion

**Figure 1:**
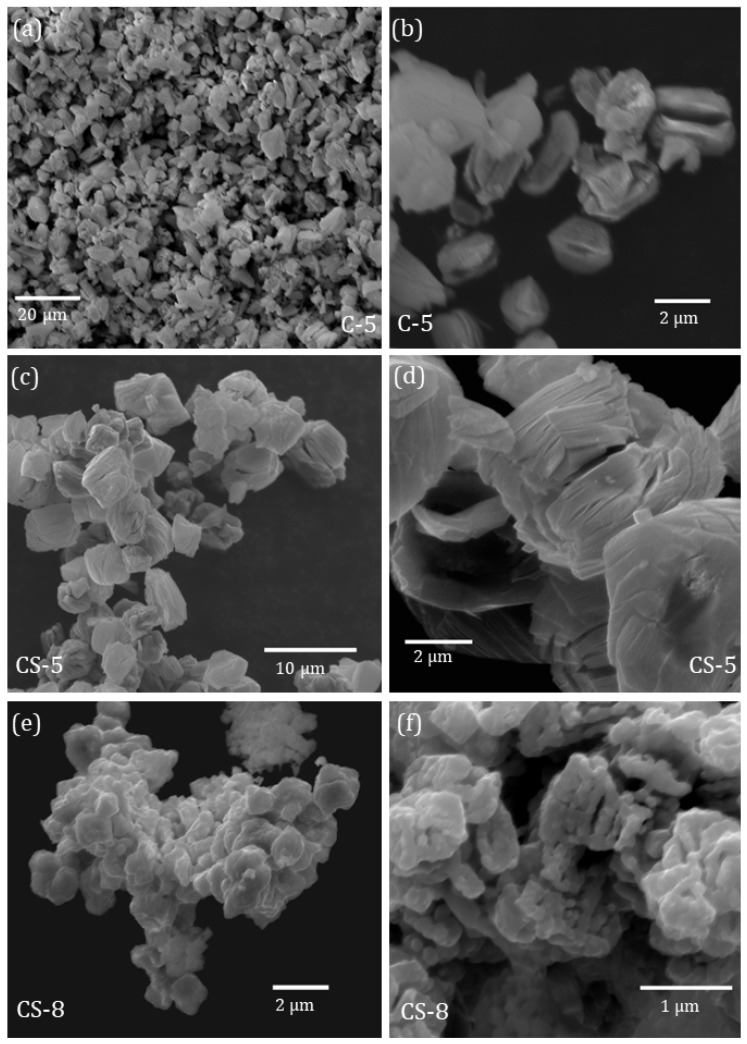
SEM images of microparticles of (**a**,**b**) C-5, (**c**,**d**) CS-5, and (**e**,**f**) CS-8 samples.

SEM analysis was employed to study the morphology and dimensions of the as-synthesised particles. All synthesised particles exhibit a rounded appearance and homogeneous sizes in the range of 2–6 μm, as shown in Figure 1. The core particles of the C-5 sample are round-shaped with dimensions of 2–3 μm, as shown in Figure 1a, although microcracks can be discerned at the surface of some particles (Figure 1b). Similar to the C-5 core sample, the core–shell microparticles of the CS-5 sample exhibit a rounded appearance but are slightly larger in size compared to the core particles, with homogeneous dimensions of around 3–5 μm (Figure 1c). The surfaces of these microparticles, smoother than those of the C-5 sample, appear occasionally covered by weak longitudinal cracks, as observed in Figure 1d, although the number of cracks is lower than for C-5. When a second thermal treatment at 800 °C is applied to these particles, as in the case of the sample CS-8, the number of surface cracks decreases, leading to particles with a grainy appearance (Figure 1e). The agglomeration of particles and their occasional irregular appearance (Figure 1f) hinder the estimation of their average dimensions, although they still remain in the range of a few microns.

EDS microanalysis confirms that the particles consist only of Ni, Mn, Co, and O, along with residual C, within the resolution of the technique. Compositional mappings of the microparticles, included as supplementary information, indicate a homogeneous distribution of elements at the microscale, without clear element segregations (Figure S1). However, local variations in their composition were observed as a function of the probed area.

**Figure 2:**
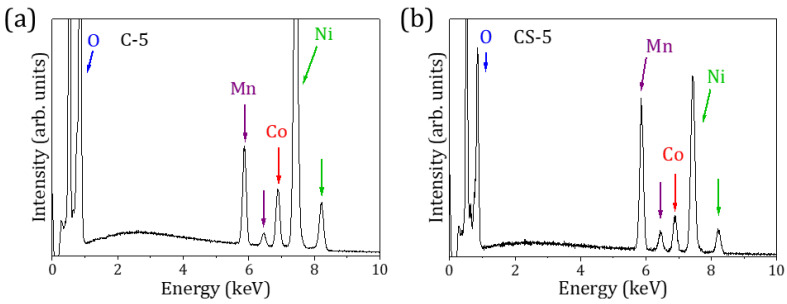
EDS spectra of (**a**) C-5 and (**b**) CS-5 samples.

Figure 2 shows the EDS spectra acquired on particles from the C-5 and CS-5 samples as representative examples of core and core–shell structures. For the C-5 sample, a larger amount of Ni in the composition is observed (Figure 2a), while the Mn/Ni ratio increases for the CS-5 core–shell sample (Figure 2b). Despite the fact that the EDS signal comes from a depth of 1–2 μm, these results point to microparticles with a Ni-rich core and a shell with an increased Mn composition, as expected. 

**Figure 3:**
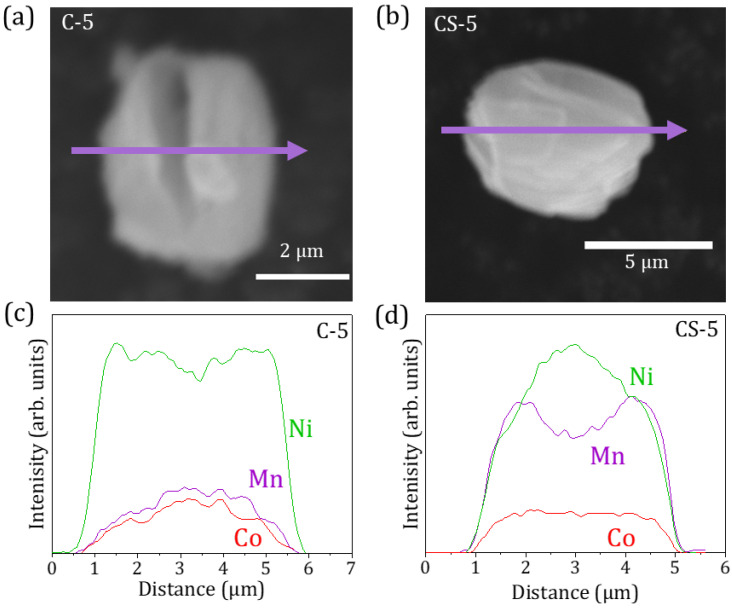
SEM images acquired on representative microparticles of samples (**a**) C-5 and (**b**) CS-5. EDS line profiles, marked with arrows in (**a**,**b**), are shown in (**c**,**d**), respectively.

In order to assess the core–shell structure based on local variations in composition, EDS line profiles were acquired for individual microparticles, as shown in Figure 3, where the SEM images of the particles and the corresponding EDS profiles are shown. Even though greater accuracy can be achieved by using complex techniques involving a focused ion beam (FIB) milling cross-sectional preparation combined with scanning-transmission electron microscopy (STEM) analysis, EDS profiles allow us to obtain insights on the core–shell structure in a simpler and more scalable way. In fact, the literature reports similar studies based on the EDS profiles of core–shell structures [27,28,29]. In this work, it should be considered that the total EDS signal decreases at the edge regions of the rounded particles due to the reduced amount of probed material in those edge areas. Therefore, larger microparticles, with dimensions of around 5 μm, were selected for this study to diminish the edge effects and facilitate the EDS analysis. The EDS signals from each element have not been corrected in Figure 3c,d; hence, attention should be paid to the compositional variations instead of the quantitative results. The at. % values after adequate corrections are included in Table S1. The compositional variations estimated from the EDS profiles, as shown in Figure 3c, confirm the homogeneous distribution of elements along the Ni-rich C-5 particles, although slight variations are observed at the edges, mainly due to the edge effect on the EDS signal. On the other hand, the CS-5 particles show a decrease in Ni content together with an increase in the amount of Mn in the edge regions, which is in agreement with a core–shell structure in which the shell exhibits a higher Mn concentration. Actually, the Mn content profile clearly shows a valley in the core region, as expected. The profile from the Co signal only shows slight variations in the edge regions of the particles owing to edge effects. Based on these EDS profiles, the averaged shell dimensions should be in the range of 0.7–1 μm, which, on average, involves approximately 35% of the radius of the particles. This value is in agreement with the increase in the average dimensions of the core–shell CS-5 microparticles as compared to the core C-5 particles, as observed by SEM. 

Regarding the CS-8 particles, the agglomeration of the particles in clusters and their irregular grainy surface hinder a conclusive analysis by EDS line profiles, although local EDS spectra acquired at diverse points of the particles also confirm the presence of a Mn-rich shell region. 

The average concentrations of Ni, Mn, and Co estimated in diverse regions of the microparticles are shown in Table 1. The atomic concentration of the sample formed by core particles (C-5) corresponds to a Ni:Mn:Co ratio of around 8:1:1, as expected. This composition is homogeneous among the numerous probed particles. However, the microparticles from the CS-5 and CS-8 samples show variations in their composition as a function of the probed region due to their core–shell structure. In this work, local EDS analysis was carried out both in the edge and microcrack regions in individual core–shell particles, the local composition of which should resemble the shell and core regions, respectively. For the core–shell particles, a higher Mn/Ni ratio is observed in the edge regions in contrast to the Ni-rich cracks, as shown in Table 1. The average Ni:Mn:Co ratio acquired in the edge regions in the core–shell microparticles slightly differs from the expected ratio of 6:3:1, as minor contributions from the Ni-rich core could be included in the total EDS signal based on the penetration depth of the technique. These results agree well with the formation of microparticles with a Mn-rich shell and a Ni-rich core. Variations in the local composition observed between the CS-5 and CS-8 samples can be due to slight differences in the average shell dimensions or the irregular shell in the CS-8 particles, which leads to larger errors in the compositional estimation. 

**Figure 4:**
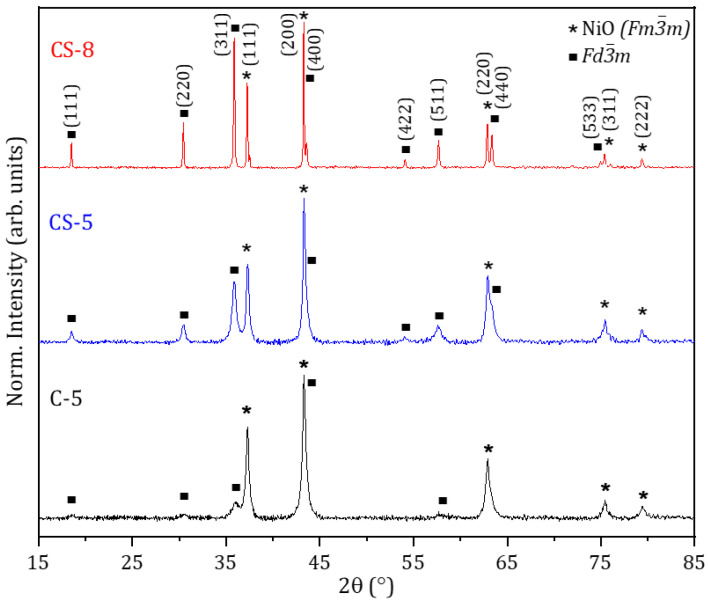
XRD patterns of C-5, CS-5, and CS-8 samples.

X-ray diffraction measurements confirm the presence of cubic NiO ($Fm\bar{3}m$) and spinel oxides ($Fd\bar{3}m$), as shown in Figure 4. Apart from NiO, the presence of other Mn and/or Co binary oxides can be disregarded in this case within the resolution of the technique. As observed in Figure 4, the main diffraction maxima from the core sample (C-5) correspond to cubic NiO, while the peaks associated with $Fd\bar{3}m$ spinel oxides exhibit a lower relative intensity. The presence of spinel oxides increases in the CS-5 and CS-8 core–shell samples, which could indicate that the shells of these microparticles should mainly be composed of spinel compounds. In particular, the relative intensity of the reflections related to spinel oxides increases for the sample treated at a higher temperature, CS-8, which also exhibits improved crystallinity based on the narrowing of the corresponding XRD maxima. In that case, the spinel signal is dominated by the (311) reflection, while the (200) peak from NiO shows a higher relative intensity. No significant shifts are observed in the XRD maxima among the different samples, which supports rather good homogeneity in the composition. 

In the analysis of the XRD patterns, it should be considered that most of the AB_2_O_4_ spinel oxides, where A and B cations correspond to Ni, Mn, and/or Co, exhibit close diffraction maxima, which hinders their univocal identification based on XRD analysis. However, some of these compounds, such as NiCo_2_O_4_ [30,31], are not stable at temperatures above 400 °C, like those used in this work. Hence, other spinel oxides such as NiMn_2_O_4_ and MnCo_2_O_4_ are considered in this work, the main diffraction maxima of which are present in the XRD patterns shown in Figure 4. Based on the EDS analysis and the increased concentration of Mn with respect to Co, mainly in the edge regions, the presence of MnCo_2_O_4_ in the microparticles should be lower than NiMn_2_O_4_. A possible mix of Ni, Mn, and Co cations in the spinel structure, the presence of other complex oxides with $Fd\bar{3}m$ structures, or Mn- and/or Co-doped NiO could also be considered in this case. 

**Figure 5:**
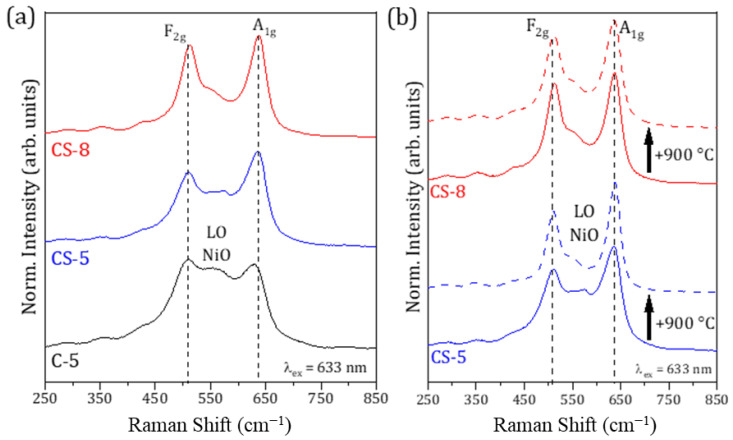
(**a**) Raman spectra of C-5, CS-5, and CS-8 samples. (**b**) Raman spectra of CS-5 and CS-8 samples acquired before (solid lines) and after (dashed lines) the thermal treatment at 900 °C.

Raman spectroscopy was employed to achieve deeper insights into the crystalline structure of the core and core–shell particles using a red laser as the excitation source (λ = 633 nm); the penetration depth should be in the micrometric range. In this case, the size of the laser spot, around 2 μm, allows for the individual study of the particles but hinders the determination of the proper location and the analysis of the edge and crack regions, as carried out by EDS. Since no large variations in the Raman signal were observed among the probed particles from each sample, the average Raman spectra are included in Figure 5 as representative results.

All the analysed particles show a dominant Raman signal in the range of 400–700 cm^−1^, as observed in Figure 5a. The main vibrational modes in this range, marked with dotted lines in Figure 5a, are centred around 510 and 640 cm^−1^. These modes can be associated with the F_2g_ (510 cm^−1^) and A_1g_ (640 cm^−1^) vibrational modes from spinel oxides, such as NiMn_2_O_4_ and MnCo_2_O_4_, which is in agreement with the XRD results. The similarity of the vibrational modes of diverse spinel Ni-Mn-Co compounds demands a detailed analysis of the Raman signal. In this study, it should be considered that the F_2g_ vibrational mode from MnCo_2_O_4_ is commonly shifted to lower wavenumbers, around 490 cm^−1^, while the A_1g_ mode is shifted to a higher wavenumber of 640 cm^−1^ [32,33], as compared to NiMn_2_O_4_. On the contrary, the vibrational modes from NiMn_2_O_4_ are more in agreement with the observed Raman signal (Figure 5). In that case, the F_2g_ mode is commonly related to Ni-O stretching modes, while the A_1g_ mode is assigned to the Mn-O asymmetric vibrations of MnO_6_ octahedral units, as reported by other authors [34,35]. In this work, the relative intensity of the F_2g_ and A_1g_ modes increases for the core–shell particles, in particular for those treated at higher temperatures, such as CS-8, which could involve microparticles with improved crystallinity, being in good agreement with the XRD results. This sharpening of the Raman modes, together with the absence of shifts in the Raman signals, points to the dominant presence of NiMn_2_O_4_ in the probed samples, mainly for the core–shell ones. 

The Raman spectra from NiMn_2_O_4_ and other Ni-Mn-Co spinel compounds usually show dominant A_1g_ vibrational modes [12,36], although in this case, a similar Raman intensity is observed for F_2g_ and A_1g_. Actually, the Raman mode F_2g_ shows a higher relative intensity for the core particles, which could be due to additional contributions to the Raman signal in that range or variations in the spinel structure inversion degree, along with variations in the Ni-O domains due to defects or the possible substitution of Ni by other cations. In this sense, a lower intensity shoulder is also observed in the Raman spectra at around 560 cm^−1^, which could be associated with LO modes in cubic NiO, although a complementary weak contribution related to E_g_ vibrational modes from spinel oxides could also be considered in this wavenumber region. The relative intensity of this LO mode decreases in the core–shell samples, mainly for CS-8, which could be related to the decreased presence of NiO in those samples, in agreement with XRD analysis. 

After the initial analysis of the Raman signal, the samples with core–shell particles (CS-5 and CS-8) were treated at 900 °C for 2 h, and then the Raman analysis was repeated under the same conditions in order to study the influence of the annealing on the evolution of the core–shell structure and the corresponding Raman signal. After the thermal treatment, both samples show Raman spectra with narrower and more intense peaks, as compared to the initial Raman spectra, and a lower relative intensity for the LO modes from NiO. This effect is clearer for the CS-5 sample, whose Raman spectrum after annealing at 900 °C resembles that of CS-8, as expected. For the sample CS-8, minor changes can be appreciated in the Raman signal after the thermal treatment, as the temperature of this annealing is similar to that employed in the synthesis process. 

Therefore, based on the Raman, XRD, and EDS results, core–shell particles should be formed by a combination of NiO and spinel oxides, where the shell region should be mainly formed by spinel oxides, in particular NiMn_2_O_4_, with a possibly lower amount of MnCo_2_O_4_. The formation of spinel oxides is also promoted by high thermal annealing. The presence of doped NiO should also be considered in this case, as well as strain effects [37]. 

**Figure 6:**
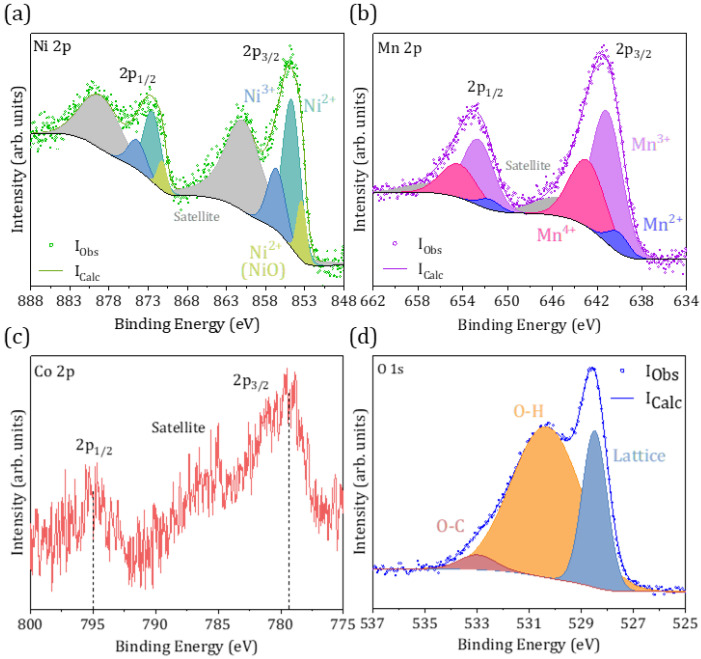
XPS spectra of CS-8 sample showing (**a**) Ni 2p, (**b**) Mn 2p, (**c**) Co 2p, and (**d**) O 1s core levels.

Finally, an XPS analysis was performed on the CS-8 sample in order to assess the electronic properties of the microparticles’ surface and determine if the shell is formed by spinel oxides either alone or in combination with NiO. Only the microparticles from CS-8 were analysed in this case, as a larger amount of spinel oxides was confirmed in this sample, especially in the shell region, based on the XRD and Raman measurements. The XPS spectra were calibrated using the C 1s peak at 284.6 eV from adventitious carbon, while Voigt functions were used during the fitting of the core levels after Shirley background corrections. Figure 6a shows the Ni 2p core level, where contributions from Ni^2+^ and Ni^3+^ associated with the spinel phases are observed at 854.8 and 856.8 eV, respectively [38,39], the former being dominant in the XPS signal. Furthermore, a lower Ni^2+^ contribution corresponding to Ni in the NiO lattice [40,41] is also observed at 853.5 eV, thus confirming the presence of this oxide on the surfaces of the probed particles. Satellite peaks are also considered in the analysis of the Ni 2p core level. The Mn 2p core level is shown in Figure 6b, where contributions at 640.2, 641.3, and 643.1 eV related to Mn^2+^, Mn^3+^, and Mn^4+^, respectively, can be observed, together with contributions due to satellite peaks at around 647 eV. These values are in agreement with those reported in other works based on NiMn_2_O_4_ [38,42,43]. In this case, the Mn^3+^ contribution is dominant, as expected in NiMn_2_O_4_, with lower contributions from Mn^4+^ and Mn^2+^ signals, as observed in Figure 6b. Table 2 shows the estimated concentration of each oxidation state after signal corrections. In the spinel NiMn_2_O_4_ lattice, Ni^2+^ and Mn^3+^ ions tend to occupy tetrahedral and octahedral positions, in agreement with the dominant oxidation states in the corresponding core levels in Figure 6. However, a low fraction of Ni can occupy octahedral sites, leading to Mn in tetrahedral positions commonly in the Mn^2+^ state in combination with Mn^4+^ to retain charge balance [44]. Ni^3+^ can also be formed to reach a charge balance in the spinel lattice, although Ni^3+^ is usually observed in NiO and is associated with the formation of nickel vacancies in this rock–salt compound [45]. Other authors reported the formation of Ni^3+^ together with oxygen deficiency when Mn^4+^ is incorporated in the NiO lattice either interstitially and/or substitutionally [46].

The Co 2p core level shows a weak signal (Figure 6c), where 2p_3/2_ and 2p_1/2_ doublets at 779.4 and 795 eV can be observed, together with a satellite contribution. The low intensity of the Co 2p signal hinders its deconvolution, although based on the asymmetry of the 2p_3/2_ contribution, a possible mixed oxidation state with Co^2+^ and Co^3+^ can be considered. The weak Co 2p core level can be related to the low presence of spinel MnCo_2_O_4_ and/or Co-doped NiO at the surface of the CS-8 particles. 

Finally, the O 1s core level shows three main contributions at 528.5, 530.4, and 533.0 eV, which can be associated with metal–oxygen bonds or lattice oxygen in spinel compounds, together with contributions commonly related to the hydroxyl group and defect sites with low oxygen coordination or oxygen deficiency, respectively [47,48].

## 3. Materials and Methods

Core and core–shell microparticles with controlled compositions were synthesised following an oxalate-assisted co-precipitation route at the German Aerospace Center (DLR—from German affiliation) in Cologne, Germany. The co-precipitation of a mixed Ni, Mn, and Co acetate solution was first performed to obtain Ni-Mn-Co oxide particles with a core composition (NMC 811) through the dropwise addition of oxalic acid solution. The corresponding acetate ratio was refined to obtain the desired Ni-rich composition of 80% Ni, 10% Mn, and 10% Co. The resulting mixture was stirred continuously at 65 °C for a duration of 45 min. In the next step, the obtained precipitates were filtered, washed with distilled water, and finally dried at 110 °C for 1 h. To obtain core–shell structures with a core (NMC 811) and shell (NMC 631) of different compositions, a two-stage synthesis approach was applied. For this purpose, the initial core particles were suspended in the mixed metallic solution with the adequate acetate ratio to achieve an Mn-rich shell composition with 60% Ni, 30% Mn, and 10% Co, and subsequently, oxalic acid was added dropwise into this solution by repeating all further steps, as in the procedure for the core synthesis. After the chemical synthesis, the particles were heat-treated at various temperatures for 2 h, leading to the final formation of Ni-Mn-Co oxide microparticles with core and core–shell structures.

As indicated in Table 3, this work deals with the analysis of one reference sample with particles with a core (C-) composition and two samples with a core–shell (CS-) structure and heat treatment at different temperatures. 

X-ray diffraction (XRD) measurements were carried out with PANalytical X′Pert Powder equipment (PANanalytical, Malvern, UK) with Bragg–Brentano geometry using Cu Kα radiation (λ = 1.541874 Å). For the morphological study, an FEI-Inspect S50 scanning electron microscope (SEM) and a ThermoFisher-Prisma E-SEM (Thermo Fisher Scientific, Waltham, MA, USA) were employed, using acceleration voltages in the range of 2–15 kV. Energy-dispersive X-ray spectroscopy (EDS) analysis was performed with a Leica 440 Stereoscan SEM (Leica, Wetzlar, Germany) equipped with a Bruker AXS 4010 detector (Bruker, Ettlingen, Germany) using an acceleration voltage of 18 kV. Raman spectroscopy measurements were performed at room temperature using a Horiba Jobin-Yvon LabRam HR800 confocal microscope (Horiba, Kyoto, Japan) with a He-Ne (λ = 633 nm) laser as the excitation source. X-ray photoelectron spectroscopy (XPS) measurements were performed at the CNR Beamline for Advanced DiCHroism (BACH beamline) [49,50] of the Elettra synchrotron facility (Trieste, Italy) using a photon energy of 1078 eV. XPS spectra were acquired using a Scienta R3000 electron energy analyser (Scienta Omicron, Taunusstein, Germany) in normal emission geometry with an energy resolution of 180 meV.

## 4. Conclusions

In summary, core and core–shell particles based on spinel Ni-Mn-Co oxides were synthesised via a cost-efficient and easily controlled co-precipitation process. For the achievement of a Ni-rich 811 core and a Mn-rich 631 shell, a two-step wet chemical synthesis approach was applied. The microparticles show homogeneous dimensions in the range 2–6 μm and controlled composition, as assessed by SEM and EDS analysis. In particular, the EDS profiles confirm shell dimensions in the range of 0.7–1 μm. Regarding the crystalline structure, NiO and spinel oxide phases are present in the microparticles, although the presence of spinel oxides, mainly NiMn_2_O_4_, is promoted in the shell as the temperature of the synthesis is increased, as confirmed by XRD and Raman spectroscopy. XPS confirms the presence of Ni and Mn with variable oxidation states in the shell region of the core–shell particles, although Ni^2+^ and Mn^3+^ are the dominant oxidation states. The controlled synthesis of the core–shell particles and the deeper knowledge of their properties widen their applicability in diverse fields of research. In particular, further research on Li incorporation in the synthesised Ni-Mn-Co oxides would allow for the production of NMC particles, which attract special interest as cathode materials for Li-ion batteries. In that case, the core–shell morphology would play an important role, providing the surface stabilisation of the Ni-rich core by forming an Mn-rich shell around it and therefore keeping the storage capabilities at a higher level as well as preventing cathode degradation.

## Figures and Tables

**Table 1:** Average Ni:Mn:Co ratios estimated from EDS analysis acquired in diverse regions in the samples C-5, CS-5, and CS-8.

Sample	Analysed Region	Ni:Mn:O
C-5	Core	8.1:1.0:0.9
CS-5	Crack	7.0:1.7:1.2
	Edge	6.7:2.5:0.9
CS-8	Crack	7.3:1.6:1.0
	Edge	6.5:2.2:1.2

**Table 2:** Average oxidation states (%) for Ni and Mn estimated from XPS analysis.

Oxidation States					
Ni^2+^ (NiO)	Ni^2+^	Ni^3+^	Mn^2+^	Mn^3+^	Mn^4+^
9.1	63.4	27.5	6.3	58.8	34.9

**Table 3:** List of samples including core (C-5) and core–shell (CS-5, CS-8) microparticles.

Sample	Structure	Treatment
C-5	Core	500 °C/2 h
CS-5	Core–shell	500 °C/2 h
CS-8	Core–shell	500 °C/2 h + 800 °C/2 h

## Data Availability

The data presented in this study are available in article and Supplementary Materials.

## Supplementary Materials

The following supporting information can be downloaded at https://www.mdpi.com/article/10.3390/molecules29122927/s1: Figure S1: SEM images of (a) CS-5 and (b) CS-8 samples and their corresponding Ni, Mn, and Co compositional mappings; Table S1: Average at. % values acquired in diverse regions of the microparticles of the C-5, CS-5, and CS-8 samples.
