# Supplementary material for: Synthesis and Characterisation of Core–Shell Microparticles Formed by Ni-Mn-Co Oxides
Source: Molecules. 2024 Jun 20;29(12):2927. doi: 10.3390/molecules29122927 (PMC11206299; doi:10.3390/molecules29122927)
Supplement: Supplementary file 1 [file molecules-29-02927-s001.zip › molecules-3040019-supplementary.pdf]

Supplementary Information:

Synthesis and Characterisation of Core–Shell Microparticles Formed by Ni-Mn-Co Oxides

Javier García-Alonso [1], Svitlana Kruger [2] , Bilge Saruhan [2] , David Maestre [1,*] and Bianchi Méndez [1]

1 Departamento de Física de Materiales, Facultad de CC. Físicas, Universidad Complutense de Madrid, 28040 Madrid, Spain; jgarci13@ucm.es (J.G.-A.)

2 Institute of Materials Research, German Aerospace Center (DLR e.V.), Linder Hoehe, 51147 Cologne, Germany

* Correspondence: dmaestre@ucm.es

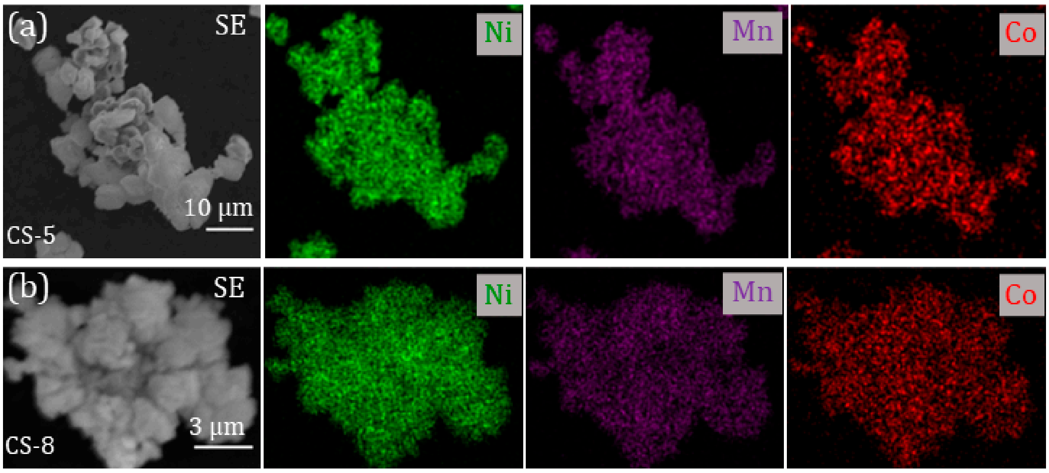

Figure S1. SEM images of (a) CS-5 and (b) CS-8 samples and their corresponding Ni, Mn, and Co compositional mappings.

Table S1: Average at. % values acquired in diverse regions of the microparticles of the C-5, CS-5, and CS-8 samples.

Sample	Analysed region	Ni (at. %)	Mn (at. %)	Co (at. %)	O (at. %)
C-5	Core	40.8 ± 1.4	4.7 ± 0.8	4.7 ± 0.8	49.8 ± 2.3
CS-5	Crack	20.7 ± 2.7	4.9 ±1.8	3.5 ± 1.5	70.8 ± 3.2
	Edge	7.4 ± 5.4	3.0 ± 4.4	1.0 ± 2.0	88.5 ± 2.6
CS-8	Crack	33.5 ± 1.6	7.5 ± 1.1	4.7 ± 0.8	49.2 ± 2.2
	Edge	23.1 ± 2.3	8.0 ± 1.7	4.6 ± 1.2	64.3 ± 2.9
